# Effects of the COVID-19 pandemic on mental healthcare and services: results of a UK survey of front-line staff working with people with intellectual disability and/or autism

**DOI:** 10.1192/bjb.2021.52

**Published:** 2022-08

**Authors:** Rory Sheehan, Christian Dalton-Locke, Afia Ali, Norha Vera San Juan, Vaso Totsika, Angela Hassiotis

**Affiliations:** 1 University College London, UK; 2 King's College London, UK

**Keywords:** COVID-19, coronavirus, intellectual disability, autism, mental health services

## Abstract

**Aims and method:**

Mental health services have changed the way they operate during the COVID-19 pandemic. We investigated the challenges and innovations reported by staff working in services for people with intellectual disability and/or autism in National Health Service (NHS) and non-NHS sectors, and in in-patient and community settings.

**Results:**

Data were drawn from 648 staff who participated in a UK-wide online survey. Issues around infection risk and mitigation were more important to those working in the NHS and in-patient settings. Community staff were more likely to express concern about the practicalities of a rapid shift to remote working and engaging patients remotely. Qualitative data revealed support for maintaining remote staff working and remote service provision post-pandemic.

**Clinical implications:**

Given the current emphasis on community support for people with intellectual disability and/or autism, the focus of research and clinical practice should be the development of accessible and effective models of remote service provision.

The COVID-19 pandemic presents specific challenges for people with intellectual disability and/or autism. People with these neurodevelopmental disorders often have co-occurring physical health conditions, including frailty, obesity, diabetes and respiratory disease,^1^ which immediately make them more vulnerable to adverse outcomes of infection with COVID-19.^2^ A substantial proportion live in congregate settings or supported housing, sharing facilities with others and being dependent on staff for aspects of their day-to-day care. Such services are prone to the rapid spread of infection between residents.^3^ In addition, people with neurodevelopmental disorders are well known to be at higher risk of mental health problems.^4^ The destabilising effects of service disruptions and breaks in routine, difficulty coping with social distancing and isolation, and lack of contact with family, friends and known trusted staff as a result of the COVID-19 restrictions further threaten the mental health of this group.^5^

The COVID-19 pandemic has challenged the provision of psychiatric care and forced a reduction in face-to-face appointments.^6^ Front-line staff working in mental health and intellectual disability services have quickly adopted new ways of working in an attempt to balance continuity of care with reduction in transmission of infection. The effects of these rapid changes on both staff and patients are, in the main, untested.

We report the experiences of staff working with people with intellectual disability and/or autism within a variety of mental health services during the first wave of the pandemic.

## Method

### Participants

Data were provided by 648 staff who worked wholly or in part with people with intellectual disability and/or autism. The majority of respondents were female (*n* = 401, 78.9%), aged 25–64 years (*n* = 471, 92.7%) and of White ethnicity (*n* = 421, 82.2%). Respondents had been working in mental health services for a mean of 14.5 years (s.d. 10.5 years). Most were based in England (*n* = 526, 81.3%), with smaller numbers in Scotland (*n* = 69, 10.6%), Wales (*n* = 39, 6%) and Northern Ireland (*n* = 6, 0.9%). One-hundred and eighty-two were nurses (28.2%), 104 were psychologists (16.1%), 55 were psychiatrists (8.5%), 40 were social workers (6.2%) and 109 were support workers (16.9%). One-third of respondents indicated that they had management responsibilities (*n* = 230, 35.5%). Just over half worked in community settings (*n* = 373, 57.6%). The majority worked in the National Health Service (NHS) (*n* = 539, 83.1%), with the remainder working in social care, voluntary or private sectors. Full demographic details, including missing data for each item, are provided as Supplementary data available at https://doi.org/10.1192/bjb.2021.52.

### Measures

Participants completed a survey comprising three main sections: (a) challenges at work during the COVID-19 pandemic; (b) staff perspectives of problems faced by mental health patients and family carers; and (c) sources of help at work in managing the effects of the pandemic. Respondents who indicated that they worked with people with intellectual disability and/or autism were filtered to an additional set of questions specifically relevant to this group. Most of the survey items were in the form of short statements which respondents rated on a five-point Likert scale between ‘not relevant/not important’ and ‘extremely relevant/extremely important’. Qualitative data on service innovations and adaptations that should be maintained after the pandemic were collected through an open-ended question.

### Procedure

Originally conceived to capture staff views across a range of mental health services, the survey was developed by clinicians, academics and people with relevant lived experience working as part of the National Institute for Health Research (NIHR) Mental Health Policy Research Unit (PRU).^7^ An expert in neurodevelopmental disorders (A.H.) provided input into questions relating to people with intellectual and other developmental disabilities. Following internal piloting, the survey was made publicly available on the Opinio platform and was disseminated via multiple channels, including the Royal College of Psychiatrists and the Royal College of Nurses, trade unions, social media, academic interest groups, housing providers, and social care and voluntary sector organisations. The survey was open during the mid-stage of the first UK national lockdown restrictions, relatively early in the pandemic (22 April to 12 May 2020). In total, 3172 individuals participated in the survey. Of these, 902 (28.4%) indicated that they worked with people with intellectual disability and/or autism. Of those, 648 had at least one valid response in each of the main sections; these form the total sample for the present study.

### Analysis

We compared survey responses between those working in the NHS and in non-NHS sectors (e.g. social care) to investigate whether challenges were experienced differently across sectors, as COVID-19 related policies and guidelines focused initially on the NHS. Similarly, we investigated potential differences between in-patient and community services, as COVID-19 policies and guidelines during the first wave were focused primarily on in-patient settings. Breakdown of each group by staff discipline is provided as Supplementary data. We excluded respondents who indicated that they worked in both in-patient and community settings, and those who worked within the NHS *and* non-NHS sectors, from this part of the analysis.

The survey included between 97 and 277 questions (depending on eligibility for branching questions) and was anticipated to take between 15 and 30 min to complete. Analyses compared whether respondents endorsed the item as ‘relevant’ to them versus ‘not relevant’. Comparisons were conducted using chi-squared tests. We applied a stringent alpha of 0.001 to avoid type 1 errors due to multiple testing. All analyses were conducted using Stata v15.^8^

Content analysis was used to analyse participants’ open-ended responses to the question ‘Has any innovation or change been made in mental healthcare that you would like to remain in place after the pandemic subsides?’ Qualitative data were analysed only by sector, as the in-patient subgroup had a small number of valid responses. Researchers initially familiarised themselves with the data and then developed codes to report the qualitative data in more succinct representative categories.^9^ To enhance validity, two researchers (V.T. and A.A.) worked in parallel to explore the unedited participant responses and identify emerging themes and subthemes, again by each subgroup, using Microsoft Excel to organise the data. The final coding frame was developed through ongoing discussion between these researchers and with the whole team, and was applied independently by the two researchers on 25% of randomly selected participant responses. Interrater reliability was good (78%); therefore, the remaining data were indexed by one researcher (V.T.). Themes are illustrated with anonymised direct quotations.

### Ethics approval and consent

The study^7^ was approved by the King's College London Research Ethics Committee (MRA-19/20-18372). Information on participation was provided on the front page of the survey. By starting the survey, participants agreed that they had read and understood all this information. It was explained on the front page of the survey that responses may be used in articles published in scientific journals, and that these articles will not include any information which could be used to identify any participant.

## Results

### NHS versus non-NHS staff

#### Challenges

Those working in NHS settings (*n* = 518) were more concerned about being infected with COVID-19 while at work than those working in non-NHS settings (*n* = 109): 84.8% rated this item as relevant compared with 70.4%, respectively (*P* < 0.001) (Table 1). NHS staff identified more problems with implementing precautions designed to minimise the transmission of infection, including: lack of personal protective equipment (65.7% *v.* 52.3%, *P* = 0.009); difficulty putting infection control measures into place (70.9% *v.* 54.6%, *P* < 0.001); and problems resulting from lack of access to testing (73.2% *v.* 56.0%, *P* < 0.001).

**Table 1:** NHS versus non-NHS sector staff (survey items with significant differences in responses)

	Survey item	Group	Not relevant, *n* (%)	Relevant, *n* (%)	χ^2^	*P*
More relevant to NHS staff	The risk I or my colleagues could be infected with COVID19 at work	NHS	78 (15.2)	434 (84.8)	12.66	<0.001
		Non-NHS	32 (29.6)	76 (70.4)		
	Having to adapt too quickly to new ways of working	NHS	20 (3.9)	496 (96.1)	19.66	<0.001
		Non-NHS	16 (14.8)	92 (85.2)		
	Having to learn to use new technologies too quickly and/or without sufficient training and support	NHS	80 (15.5)	436 (84.5)	20.11	<0.001
		Non-NHS	37 (33.9)	72 (66.1)		
	Being expected to use new technologies without reliable access to necessary tools and equipment	NHS	108 (21.0)	407 (79.0)	24.29	<0.001
		Non-NHS	47 (43.5)	61 (56.5)		
	Difficulty putting infection control measures into practice in the setting I work in	NHS	150 (29.1)	366 (70.9)	10.93	<0.001
		Non-NHS	49 (45.4)	59 (54.6)		
	Problems resulting from lack of access to testing	NHS	138 (26.9)	376 (73.2)	12.69	<0.001
		Non-NHS	48 (44.0)	61 (56.0)		
	Pressure to accept redeployment to a setting where I don't feel happy to work	NHS	348 (67.4)	168 (32.6)	11.08	<0.001
		Non-NHS	91 (83.5)	18 (16.5)		
	Difficulty maintaining adequate support for families looking after a child/young person/adult with ID and/or autism who displays challenging behaviour	NHS	97 (30.6)	220 (69.4)	12.89	<0.001
		Non-NHS	35 (53.9)	30 (46.2)		
	Guidance from my employer on managing clinical and safety needs due to COVID-19	NHS	12 (2.3)	506 (97.7)	25.11	<0.001
		Non-NHS	14 (12.8)	95 (87.2)		
	New initiatives in NHS mental health services	NHS	49 (9.6)	462 (90.4)	21.93	<0.001
		Non-NHS	28 (25.9)	80 (74.1)		
	Being aware of public support for key workers	NHS	48 (9.3)	468 (90.7)	23.07	<0.001
		Non-NHS	28 (25.9)	80 (74.1)		

ID, intellectual disability.

Having to adapt too quickly to new ways of working (96.1% *v.* 85.2%, *P* < 0.001), having to use new technologies without adequate training or support (84.5% *v.* 66.1%, *P* < 0.001), and not having necessary tools or equipment to facilitate remote working (79.0% *v.* 56.5%, *P* < 0.001) were all rated as significantly more relevant by NHS staff compared with those working in other sectors. Those working in the NHS were also significantly more likely to endorse public support for keyworkers as an important source of support than those in other sectors (90.7% *v.* 74.1%, *P* < 0.001).

#### Service innovations to maintain after the pandemic

Open-ended responses were provided by 364 participants (Table 2). Three main themes emerged from the content analysis: remote operation, flexibility and organisational improvement.

**Table 2:** Innovations and changes made during the COVID-19 pandemic that respondents would like to keep after the pandemic is over, content analysis by sector

	Sector	
	NHS	Non-NHS
Valid answers, *n*	314	50
Remote working	48%	36%
Remote services	51%	42%
Flexibility	7%	8%
Organisational improvement	17%	18%
New services	7%	4%
Staff well-being	6%	4%
Service integration	1%	2%
Better leadership	1%	2%

Remote operation included remote staff working and remote service provision as two distinct subthemes. These subthemes referred to the ability to work from home and using remote technology to communicate, provide therapy and maintain contact with clients. Remote operation was considered positive because it was more immediate, efficient, improved work–life balance and had environmental benefits. Respondents proposed that remote services for those in need reduced waiting times and the rate of non-attendance.

‘*As a carer, more home working has given me a better work–life balance…the reduced stress means I am able to focus more on patient needs*’

‘*Telephone and online counselling for some clients has been beneficial as they struggle to access the building…my DNA [did not attend] rate has decreased as a result*’

As shown in Table 2, NHS staff tended to endorse both subthemes more compared with staff from other sectors, particularly in the case of remote staff working: 48% of NHS staff identified this as a desirable innovation to maintain compared with 36% staff from other sectors. One possible reason for this difference may be that NHS staff were more likely to be able to work from home, delivering assessments, therapy and reviews remotely, whereas this was less available to those in the care sectors whose role demands face-to-face interaction.

A related theme was that of flexibility in the way professionals worked, and in the services offered to patients, including providing a choice between ‘in-person’ or remote consultations. Flexibility involved managers monitoring staff productivity by task and outcome, rather than hours spent in the office. Flexibility was thought to improve productivity, resulting in more efficient working.

‘*Patients should have the option to have a remote or face to face assessment/ sessions.*’

‘*[I've noticed] a change in attitude towards agile working – task oriented rather than a focus on time spent working*’

Organisational improvement referred to specific descriptions of organisational changes that participants identified; these included more time spent in supervision and more reflection time. Other positives were a perception of improved communication between professionals, and organisational processes that were speedier and more efficient (more use of triage, fewer meetings), as well as less reliance on in-patient admission together with a greater willingness to discharge patients There was also felt to be a renewed or new focus on what the service/staff should be doing (e.g. compassion therapy, being nice to each other, providing therapy to clients). Within this theme, staff across sectors reported a range of ideas about new services they would like to see continued, including the integration of existing services, initiatives focused on staff well-being, and changes in the communication and leadership style of those in management positions.

‘*Sharing of updates when they happen, ironing out issues immediately*’

‘*More thinking outside the box, how staff can facilitate activities, more hands on approach*’

‘*The pandemic has seen a shift from people queuing up for informal admissions and not wanting to be discharged when in hospital to hardly anyone wanting/saying they need to be in hospital*’

‘*Colleagues being very caring and looking after one another…the team has really pulled together for clients’ and staff well-being*’

‘*E-mails from senior managers and directors have taken a much more humane tone, and seem far more genuine than the usual bland, generic, corporate speak*’

### Community versus hospital in-patient staff

Staff working in hospital in-patient settings (*n* = 96) were more concerned about infection risk to themselves and to their family/friends through them, and about the spread of infection between patients compared with those working in community settings (*n* = 265) (corresponding percentages 96.9% *v.* 72.0%, 94.8% *v.* 63.8%, 100% *v.* 54.6%, respectively, all *P* < 0.001) (Table 3). Staff in in-patient settings had greater problems implementing infection control measures, including lack of personal protective equipment (PPE) and lack of access to testing. In-patient staff were more likely to report feeling under pressure from managers or colleagues to be less cautious about infection control than they would like to be (60.0% *v.* 31.7%, *P* < 0.001) and were more likely to express problems related to working with non-permanent colleagues (i.e. redeployed, agency or locum staff).

**Table 3:** Community versus hospital in-patient staff (survey items with significant differences in responses)

	Survey item	Group	Not relevant, *n* (%)	Relevant, *n* (%)	χ^2^	*P*
More relevant to in-patient staff	The risk I or my colleagues could be infected with COVID19 at work	In-patient	3 (3.1)	93 (96.9)	25.85	<0.001
		Community	73 (28.0)	188 (72.0)		
	The risk family or friends may be infected with COVID19 through me	In-patient	5 (5.2)	91 (94.8)	33.65	<0.001
		Community	96 (36.2)	169 (63.8)		
	The risk that COVID19 will spread between patients I'm working with	In-patient	0 (0)	96 (100)	65.31	<0.001
		Community	119 (45.4)	143 (54.6)		
	Difficulty putting infection control measures into practice in the setting I work in	In-patient	9 (9.4)	87 (90.6)	36.98	<0.001
		Community	115 (43.9)	147 (56.1)		
	Lack of protective clothing (PPE) and equipment needed for infection control	In-patient	21 (21.9)	75 (78.1)	16.15	<0.001
		Community	119 (45.3)	144 (54.8)		
	Problems resulting from lack of access to testing	In-patient	12 (12.6)	83 (87.4)	20.41	<0.001
		Community	99 (37.6)	164 (62.4)		
	Feeling under pressure from managers or colleagues to be less cautious about infection control than I would like	In-patient	38 (40.0)	57 (60.0)	23.51	<0.001
		Community	181 (68.3)	84 (31.7)		
	Not enough of the team I'm working with are permanently employed in this setting	In-patient	33 (34.4)	63 (65.6)	47.61	<0.001
		Community	196 (74.0)	69 (26.0)		
More relevant to community staff	Having to learn to use new technologies too quickly and/or without sufficient training and support	In-patient	22 (22.9)	74 (77.1)	11.31	<0.001
		Community	25 (9.4)	240 (90.6)		
	Increased difficulty managing work–life balance	In-patient	51 (53.1)	45 (46.9)	10.70	0.001
		Community	90 (34.1)	174 (65.9)		
	Difficulty engaging people with ID and/or autism with remote appointments	In-patient	30 (45.5)	36 (54.6)	18.60	<0.001
		Community	31 (18.1)	140 (81.9)		
	Difficulty maintaining adequate support for families looking after a person with ID and/or autism who displays challenging behaviour	In-patient	34 (50.8)	33 (49.3)	13.24	<0.001
		Community	44 (26.0)	125 (74.0)		

ID, intellectual disability.

Staff working in the community reported greater difficulty in adapting to using new technologies in their work and in engaging patients with neurodevelopmental disorders in remote appointments (54.6% *v.* 81.9%, *P* < 0.001). They also expressed more difficulty in managing their work–life balance (65.9% *v.* 46.9%, *P* = 0.001).

## Discussion

### Main results and implications

This is one of the first studies to examine the experiences of front-line staff working with people with intellectual disability and/or autism during the COVID-19 pandemic. The survey data were collected at the peak of the first wave, when national lockdown restrictions were at their height and there was considerable uncertainty and anxiety around individual outcomes of infection and the implications of the pandemic for healthcare services and for society as a whole.

Exploring differences in responses between staff working in different settings and across sectors provides insight into what matters, where and to whom. This understanding can inform targeted service delivery and resource allocation that will increase resilience during subsequent waves of the pandemic or future public health emergencies.

Those working in in-patient settings were particularly concerned about the risk of being infected with COVID-19. Preventing infection spread on hospital wards is likely to be challenging given the close and prolonged contact between staff and patients and considering that people with intellectual disability and/or autism may have difficulties understanding and adhering to social distancing advice. There were also concerns from in-patient staff about insufficient measures to mitigate spread of infection (PPE and access to testing), issues which remain alive several months into the pandemic. Conversely, those working in community settings were more likely to rate problems with using remote technology as more relevant, probably reflecting the potential for them to work remotely in a way not possible for in-patient staff. Difficulties associated with remote working included inadequate training and support, perhaps reflecting the pace and scale of the change demanded by the pandemic. Furthermore, the loss of demarcation between home and work spaces when working from home is likely to underlie a reported negative effect on work–life balance in those working in community posts.^10^ These differences (among others shown in Table 3) highlight the need to tailor staff support to different groups.

Those working in the NHS were more likely than those in other sectors to express problems with having to adapt too quickly to new ways of working, having insufficient support to use new technologies, and lacking necessary tools and equipment to make remote working a reality. This could represent a lack of preparedness and agility in NHS trusts, given that they are likely to be large organisations with multiple layers of management and centralised information technology (IT) support and procurement services. Experiences during the pandemic may be used to stimulate greater investment in IT infrastructure that will support the digital transformation hailed in the NHS Long Term Plan.^11^

Despite these difficulties, remote delivery of services to people with intellectual disability and/or autism was widely endorsed by staff as one of the main innovations and service changes to be maintained post-pandemic. Although this was more frequent among staff in the NHS, staff in other sectors also often identified the benefits and flexibility of remote service operations, e.g. in carrying out a variety of care assessments. ‘Telemental health’, although available for several years prior to the pandemic, had not been widely adopted by the NHS,^12^ despite some benefits having been shown in terms of efficiency and flexibility.^13^ Unless there is a widening remit of its application, buy-in by providers and adaptation to facilitate use by this population group, the efforts to ‘digitise’ during the pandemic will be a missed opportunity to achieve lasting positive change. Maintaining dialogue between stakeholders and following a co-production approach to the further development of remote services will be important to ensure efficient and equitable care.

Staff health and well-being has been a concern throughout the pandemic. McMahon et al demonstrated moderate levels of burnout and mild-to-moderate levels of anxiety and depression in a cohort of staff working in intellectual disability services in Ireland during the pandemic,^14^ providing further evidence of the array of adverse psychosocial effects on staff working through the pandemic.^15–18^ In response to these pressures, a number of initiatives and interventions have been made available to increase staff coping, including ‘care packages’ (consisting of essentials and treats for staff), online therapy or counselling, and resources to manage depression, anxiety or insomnia.^19^ Our results show that in addition to short-term interventions focused on the individual, broad organisational and cultural shifts, including a ‘*less authoritarian and more collaborative*’ style from senior managers, were valued by front-line staff. As the pandemic stretches into the future, and with the threat of further waves (possibly caused by mutant viral strains), it will be important to shift from crisis management to embedding new ways of working. This process should include seeking and listening to the views of front-line staff in determining what is important to them,^20^ and including them in formal evaluations of new models of working, if we are to ensure a capable and resilient workforce who are enabled to continue to provide first-class care.

### Limitations

This is a secondary analysis of data from a larger survey and shares the limitations of the original study.^7^ It presents the perspective of staff and, as such, is only one viewpoint, albeit a direct one, of what have been unprecedented changes. The sample was one of convenience: although the survey was widely advertised and respondents were drawn from a number of different sources, it may not be representative of all staff working in mental healthcare for people with intellectual disability and/or autism. Staff in the present sample worked with people with intellectual disability and/or autism but could also work with other patient groups, and it was not possible to disentangle which opinions are informed by work with specific groups; however, their experiences are overarching, as many ‘mainstream’ mental health services are accessed by people with neurodevelopmental disorders. Differences in survey responses between staff working in different sectors and settings may, in part, reflect differences in the distribution of professional roles and duties within these groups.

Survey responses were gathered during an extraordinary period and reflect the challenges at that time. Respondents working in the NHS were overrepresented compared with those working in other sectors, and those working in community settings outnumbered those working in in-patient units. The questionnaire, while broad and devised by a multidisciplinary team including experts by experience, was necessarily rapidly developed, and it is possible that some important aspects of the pandemic and its effects on mental health services were not covered. Many other professionals important to the mental healthcare of people with intellectual disability and/or autism, such as those working in primary care and pharmacists, were not included in the sample. Future work should also aim to increase participation rates of non-White staff groups, given what is now known about the effects of COVID-19 on their overall health and their importance as key workers.^21^ Informal carers for people with intellectual disability are a further group who may experience disproportionate levels of stress and mental health problems.^22^ It is also necessary to hear directly from patients in order to understand their experience of mental healthcare and to ensure services are responsive to their needs.

### Summary

Staff who worked with people with intellectual disability and/or autism during the first wave of the COVID-19 pandemic identified challenges related to infection control and to adapting to new, mostly remote, ways of working. As the pandemic continues, staff need to be supported by their employers to feel safe and to minimise the risk of acquiring or transmitting COVID-19, and with interventions and initiatives that maintain their well-being and reduce the risk of burnout. Staff believe that remote service provision to people with intellectual disability and/or autism is both possible and desirable; the challenge to our specialty is the development of accessible and effective models of remote care.

## Data Availability

The survey dataset is currently being used for additional research and is therefore not currently available in a data repository. A copy of the survey is available at this web address: https://opinio.ucl.ac.uk/s?s=67819.

## About the authors

**Rory Sheehan** is an Honorary Research Fellow at the Division of Psychiatry, University College London, London, UK; **Christian Dalton-Locke** is a Research Assistant at the Division of Psychiatry, University College London, London, UK; **Afia Ali** is an Associate Professor at the Division of Psychiatry, University College London, London, UK, and an Honorary Consultant Psychiatrist at the North East London NHS Foundation Trust; **Norha Vera San Juan** is a Research Associate at the Health Service and Population Research Department, Institute of Psychiatry, Psychology and Neuroscience, King's College London, London, UK; **Vaso Totsika** is an Associate Professor at the Division of Psychiatry, University College London, London, UK; **Angela Hassiotis** is a Professor at the Division of Psychiatry, University College London, London, UK, and an Honorary Consultant Psychiatrist at the Camden and Islignton NHS Foundation Trust.

## Supplementary material

For supplementary material accompanying this paper visit http://doi.org/10.1192/bjb.2021.52. click here to view supplementary material
