# Supplementary material for: Effects of the COVID-19 pandemic on mental healthcare and services: results of a UK survey of front-line staff working with people with intellectual disability and/or autism
Source: BJPsych Bull. 2022 Aug;46(4):201–7. doi: 10.1192/bjb.2021.52 (PMC9768507; doi:10.1192/bjb.2021.52)
Supplement: Supplementary file 1 [file S2056469421000528sup001.docx]

**Supplementary data table 1** Demographic and employment characteristics of survey respondents (*n*=648)

| **Demographic variables** | | | |
| --- | --- | --- | --- |
|  |  | *n* | Valid % |
| Sex | Male | 96 | 18.9 |
|  | Female | 401 | 78.9 |
|  | Other | 2 | 0.4 |
|  | Prefer not to say | 9 | 1.8 |
|  | Missing | 140 | - |
| Age group | <25 | 21 | 4.1 |
|  | 25-34 | 127 | 25.0 |
|  | 35-44 | 90 | 17.7 |
|  | 45-54 | 155 | 30.5 |
|  | 55-64 | 99 | 19.5 |
|  | ≥65 | 3 | 0.6 |
|  | Prefer not to say | 13 | 2.6 |
|  | Missing | 140 | - |
| Ethnic group | White | 421 | 86.6 |
|  | Asian | 26 | 5.3 |
|  | Black | 12 | 2.5 |
|  | Multiple groups / mixed | 20 | 4.1 |
|  | Other | 4 | 0.8 |
|  | Prefer not to say | 3 | 0.6 |
|  | Missing | 162 | - |
| Caring for children <18 years | Yes | 158 | 31.1 |
|  | No | 350 | 68.9 |
|  | Missing | 140 | - |
| Caring for elderly or disabled relative | Yes | 135 | 26.8 |
|  | No | 369 | 73.2 |
|  | Missing | 144 | - |
| **Employment variables** | | | |
| Work sector* | NHS | 539 | - |
|  | Social care | 52 | - |
|  | Voluntary | 43 | - |
|  | Community or user-led | 11 | - |
|  | Private | 28 | - |
| Work setting* | Hospital in-patient | 161 | - |
|  | Crisis house in community | 7 | - |
|  | Residential / supported accommodation | 40 | - |
|  | Crisis team, liaison team, AMHP team | 108 | - |
|  | Community team | 373 | - |
|  | Day/drop-in/recovery college | 51 | - |
|  | Other | 99 | - |
| Professional role | Psychologist | 104 | 16.1 |
|  | Nurse | 182 | 28.2 |
|  | Occupational therapist | 42 | 6.5 |
|  | Other qualified therapist | 75 | 11.6 |
|  | Peer support worker | 21 | 3.3 |
|  | Psychiatrist | 55 | 8.5 |
|  | Social worker | 40 | 6.2 |
|  | Manager, no mental health professional qualification | 18 | 2.8 |
|  | Other worker with direct contact with people with mental health problems | 109 | 16.9 |
|  | Missing | 2 | - |
| Managerial responsibility | Yes | 230 | 35.5 |
|  | No | 418 | 64.5 |
|  | Missing | 0 | - |
| Which country do you work in | England | 526 | 81.3 |
|  | Northern Ireland | 6 | 0.9 |
|  | Scotland | 69 | 10.7 |
|  | Wales | 39 | 6.0 |
|  | Other | 7 | 1.1 |
|  | Missing | 1 | - |
| What locality do you work in | City/town >100,000 population | 437 | 67.8 |
|  | Town < 100,000 population | 142 | 22.0 |
|  | Rural | 66 | 10.2 |
|  | Missing | 3 | - |

*Percentages not given as respondents were able to select more than one response

**Supplementary data table 2** Detail of respondents in NHS and non-NHS groups by profession or role

|  | **Work sector** | |
| --- | --- | --- |
| **Profession or role** | **NHS** *n* (valid %) | **Non-NHS** *n* (valid %) |
| Clinical or counselling psychologist | 89 (17.2) | 7 (6.4) |
| Nurse | 176 (34.1) | 6 (5.5) |
| Occupational therapist | 37 (7.2) | 5 (4.6) |
| Other qualified therapist | 67 (13.0) | 5 (4.6) |
| Peer support worker | 13 (2.5) | 7 (6.4) |
| Psychiatrist | 52 (10.1) | 1 (0.9) |
| Social worker | 18 (3.5) | 20 (18.3) |
| Manager | 5 (1.0) | 12 (11.0) |
| Other | 59 (11.4) | 46 (42.2) |
| **Total** | **516*** | **109** |

*Two missing answers

**Supplementary data table 3** Detail of respondents in in-patient and community groups by profession or role

|  | **Work setting** | |
| --- | --- | --- |
| **Profession or role** | **In-patient** *n* (valid %) | **Community** *n* (valid %) |
| Clinical or counselling psychologist | 7 (7.3) | 58 (22.0) |
| Nurse | 44 (45.8) | 72 (27.3) |
| Occupational therapist | 13 (13.5) | 15 (5.7) |
| Other qualified therapist | 5 (5.2) | 53 (20.1) |
| Peer support worker | 3 (3.1) | 7 (2.7) |
| Psychiatrist | 7 (7.3) | 14 (5.3) |
| Social worker | 1 (1) | 11 (4.2) |
| Manager | 1 (1) | 2 (0.8) |
| Other | 15 (15.6) | 32 (12.1) |
| **Total** | **96** | **264*** |

*One missing answer
